# *Moringa oleifera* Lam. Commercial Beverages: A Multifaceted Investigation of Consumer Perceptions, Sensory Analysis, and Bioactive Properties

**DOI:** 10.3390/foods12112253

**Published:** 2023-06-02

**Authors:** Jéssica Ferreira Rodrigues, Cristina Soares, Manuela M. Moreira, Maria João Ramalhosa, Neimar Freitas Duarte, Cristina Delerue-Matos, Clara Grosso

**Affiliations:** 1 Departamento de Ciência dos Alimentos#x2014;DCA/UFLA, Universidade Federal de Lavras, Lavras 37200-000, Brazil; 2 REQUIMTE/LAQV (Network of Chemistry and Technology/Associated Laboratory for Green Chemistry), Instituto Superior de Engenharia do Porto, Instituto Politécnico do Porto, Rua Dr. António Bernardino de Almeida 431, 4249-015 Porto, Portugal; manuela.moreira@graq.isep.ipp.pt (M.M.M.); mjr@isep.ipp.pt (M.J.R.); cmm@isep.ipp.pt (C.D.-M.); claragrosso@graq.isep.ipp.pt (C.G.); 3 Department of Agrarian Sciences, Federal Institute of Minas Gerais (IFMG—Santa Luzia), Santa Luzia 33115-390, Brazil; neimar@ifmg.edu.br

**Keywords:** consumer perceptions, sensory attributes, health claims, phytochemical composition, antioxidant activities, chemovariation, beverage acceptance, heavy metal contaminants

## Abstract

This study employs a multidisciplinary approach to evaluate consumers’ perceptions and acceptance of *Moringa oleifera* Lam. beverages, examining sensory attributes, chemical composition, and bioactivities. High-performance liquid chromatography with diode array detection (HPLC-DAD) analyses revealed significant chemovariation in phenolic compositions among commercial moringa beverages. A soluble moringa powder drink exhibited the greatest concentrations of phenolic and flavonoid compounds, along with powerful antioxidant capacity powers assessed with ABTS^•+^, DPPH^•^, FRAP assays, ^•^NO, and H_2_O_2_ scavenging activities. However, this sample was the least preferred and presented high Cd levels, exceeding WHO-acceptable values of 0.3 mg/kg. Sensory testing indicated that sweet and floral flavors contributed to beverages being liked, while green, grass, herbal flavors, sour, bitter, and precipitate presence were considered unfavorable sensory attributes. Health claims positively influenced acceptance, particularly among women. Consumers associated feelings of health, wellness, relaxation, and leisure with moringa beverages. During purchase, the most observed information included the ingredient list, health benefits, and type/flavor. These findings emphasize the importance of consumer awareness in reading labels, verifying product origins, and ensuring the absence of contaminants. By understanding consumer preferences and the impact of health claims, producers can better tailor *M. oleifera* beverages to meet consumer expectations while maintaining safety and quality standards.

## 1. Introduction

For millennia, people have been enjoying tea and it is one of the most popular beverages due to its flavor and health benefits [1]. Herbal teas prepared from other plant species are also greatly consumed. Among several herbal beverages available on the market, moringa decoction is paving its way to high popularity due to its health-related benefits, usually attributed to its antioxidant and phenolic contents.

Moringa decoction is made from the leaves of *Moringa oleifera* Lam., a persistent deciduous plant from the *Moringaceae* family that thrives in tropical conditions. *M. oleifera* has been considered a “Miracle tree” and a “Tree of life” due to its considerable favorable effects on health, nutrition, water sanitation, and the environment [2]. Attention has also been paid to its many pharmacological characteristics, such as anti-cancer, anti-diabetic, neuroprotective, anti-inflammatory, and antioxidant effects, with it being considered a functional food [3,4]. These properties lead to increased popularity for moringa decoction, with marketing strategies focused on its functional benefits. However, the decoction’s chemical composition changes dramatically during post-harvest processing, and is highly variable depending on the geographical origin. Therefore, commercial decoctions with diverse organoleptic qualities can be produced and traded worldwide [5]. Indeed, in Brazil, numerous products labeled as or constituted by *M. oleifera* have been irregularly marketed and disseminated with several therapeutic claims, such as being cancer cures or treatments for diabetes and cardiovascular diseases, and with advertised benefits that are not allowed for food products according to the country’s legislation. Given this, the manufacture, import, commercialization, advertising, and distribution of all foods containing *M. oleifera* were banned in Brazil. As a result of this decision, a Call Notice was opened to collect data and information on *M. oleifera* safety for use in food to support the Agency’s decision as to whether or not to maintain the health protection measures determined by Resolution-RE n. 1478, of 3/6/2019 [6]. Another concern associated with *M. oleifera* decoctions consumption is the high concentration of heavy metals, such as lead (Pb) and cadmium (Cd), absorbed by the plant from the soil, leading to a variation in heavy metal content in the final products. The origin of plants is crucial to consider, as environmental risks associated with heavy metals such as Cd and Pb are dictated by both their concentrations and the adsorption capacities of the respective soils [7]. In addition, the brewing process, which involves high temperatures, enhances the release rate of these and other harmful elements transferred from the soil to the tea leaves [8]. In fact, with the prolonged consumption of contaminated products, some elements’ cumulative toxicity might be observed in humans [9]. It is well-recognized that Cd toxicity is associated with impairments in lung, kidney, liver, skeletal, reproductive, and cardiovascular functions [10]. Exposure to Pb can lead to neurological and hematological malfunctions, as well as to damage to the kidneys and liver, and reproductive disorders in the human body [10]. Contrary to Brazil’s decision, *M. oleifera* dry leaves are sold in specialized herbal stores in Europe, with the leading market destinations being the United Kingdom, Germany, and France [11]. In Europe, *M. oleifera* is sold chiefly as capsules in the nutraceutical sector and in powder, leaf, seed, and oil forms in the food sector. The cosmetic industry also utilizes moringa oil due to its moisturizing, cleansing, and emollient properties [11]. It is also applied in aromatherapy, blends well with other essential oils, and presents good carrier oil properties [12]. The European Food Safety Authority (EFSA) has produced a technical report against the trade of products containing *Moringa stenopetala* (Baker f.) Cufod., a species native to South Ethiopia [13].

Sustainability issues are one of the main concerns of modern societies. Consumers are aware of the necessity of reducing their carbon footprint by using more natural and functional food products, while taking advantage of their health-promoting properties [14]. This not only forces rigid policy-making decisions to be established, but also makes marketing campaigns provide information to the consumers. One available strategy for companies and organizations to assess the quality of marketed products is to improve their promotional and consciousness-raising tactics by examining consumers’ perceptions and attitudes towards the properties of foods and their health benefits or harms, especially concerning interpreting and comprehending the information on food labels and nutritional claims [15]. In addition, consumers must have a particular level of understanding about nutrition and dietary recommendation guidelines to support their food choices [16]. Thus, adopting a targeted strategy to address consumer interests can enhance the overall awareness and knowledge of the benefits associated with tea and other beverages [17].

According to the Dietary Supplement Label Database of the National Institutes of Health (NIH), 376 *M. oleifera*-based supplements are available. In Europe, the European Food Safety Authority (EFSA) has only expressed concerns about the safety of *M. stenopetala*, allowing the sale of *M. oleifera* [13]. Indeed, various *M. oleifera* products from different suppliers and origins have been found in several Portuguese herbal stores. However, due to concerns about the overconsumption of *M. oleifera* in Brazil, authorities have prohibited its sale despite its beneficial effects. It is worth mentioning that even though the plant is banned in Brazilian supermarkets and herbal stores, consumers can still purchase various *M. oleifera*-based products through international websites. Conducting more studies to assess the potential of this species could provide new knowledge that may help Brazilian authorities implement guidelines for *M. oleifera* trade and consumption instead of complete prohibition, as consumers can still access these products online. Since most existing studies primarily focus on the health benefits of *M. oleifera*, this study aims to address previously unexplored topics such as the quality and variability of the composition of beverages available on the market, as well as consumer perceptions and the influence of moringa health-promoting claims. The results of this study could contribute to a better understanding of this plant species, ultimately assisting Brazilian regulatory authorities in making informed decisions about medicinal plant commercialization and guiding marketing strategies and consumer awareness.

Considering that herbal infusions’ quality and sensory characteristics have an evident impact on consumer acceptance, the present study hypothesized that health-promoting claims could influence consumers’ perceptions of the sensory attributes of *M. oleifera* beverages. Aiming to increase knowledge about moringa supplements and provide reliable scientific information that can help regulatory authorities regulate moringa commercialization, this study characterized the phytochemical composition and bioactivities of moringa beverages prepared with commercial samples of dried moringa leaves available on the market. Additionally, the evaluation considered their safety regarding heavy metal content and organoleptic characteristics, as well as the taster panel’s response to the reported health claims.

## 2. Materials and Methods

### 2.1. Reagents and Standards

Reagents for antioxidant activities and cholinesterase inhibition assays were from Sigma-Aldrich (St. Louis, MO, USA and Steinheim, Germany), except magnesium chloride hexahydrate, which was bought from VWR (Leuven, Belgium), sodium chloride from Fisher Scientific (Fair Lawn, NJ, USA), sodium nitroprusside dihydrate from Fluka, and sodium carbonate from Merck. Hydrogen peroxide 35.5% *w*/*w* was purchased from Labchem (Zelienople, PA, USA). Methanol Chromasolv for HPLC was from Riedel-de Haën (Seelze, Germany), and formic acid was from Carlo Erba (Val de Reuil, France). The standards used for HPLC analyses were acquired from Sigma-Aldrich (4-O-caffeoylquinic acid, quercetin-3-O-rutinoside hydrate, protocatechuic acid, ferulic acid, and p-coumaric acid), Fluka (caffeic acid), Alfa Aesar (5-O-caffeoylquinic acid), and Extrasynthèse (Genay, France): 3-O-caffeoylquinic acid, apigenin-8-C-glucoside, apigenin-6-C-glucoside, quercetin-3-O-glucoside, kaempferol-3-O-glucoside, kaempferol-3-O-rutinoside, and tiliroside. Ultrapure water (with resistivities of 18.2 MΩ/cm) was obtained using a Milli-Q water purification system from Millipore (Molsheim, France). Standard solutions of Pb and Cd, Atomic Absorption standard with 4% HNO_3_, were purchased from SPC Science (Quebec, Canada). Suprapur nitric acid 65% (*v*/*v*) and NH_4_H_2_PO_4_ (Suprapur) were from Merck (Darmstad, Germany).

### 2.2. Plant Material

Five commercial samples of *M. oleifera* with different origins, available on the Portuguese and Brazilian markets, were acquired for the study. Sampling was performed by searching natural products stores for dried moringa samples ready to use. Sample 1 (Brazil) was acquired in Bambuí, and the other four samples were acquired from three different natural products stores from Porto, Portugal. Samples 1, 2 (Brazil), and 5 (Egypt) were dried moringa leaves; Sample 3 (non-European origin) was acquired as a dried powder, and Sample 4 (Sri Lanka) as “tea” bags. For each sample, at least 150 g was purchased. These samples were used to perform chemical, biological, and sensory analyses. The samples were acquired within the validity period of one to two years and accompanied by a technical report from the supplier. Three of the samples hade claims on their labels, such as “miracle tree” (Sample 4), “superfood” (Sample 3), and “antioxidants and amino acids” (Sample 4). In contrast, the other two samples were described as a source of “minerals (chromium, calcium, iron, magnesium, phosphorus, vitamins, zinc and riboflavin)” (Samples 3 and 5). In Europe, moringa products are only available as food supplements.

### 2.3. Extract Preparation

The aqueous extracts of *M. oleifera* (Samples 1, 2, 4, and 5) were prepared according to the packaging specifications, by combining 1 g of plant material with 250 mL of water and heating it to 100 °C for a duration of 5 min. Sample 3 (moringa powder) was prepared according to the packaging instructions, diluting 1 g of soluble sample in 250 mL of water. After filter paper filtration, the extracts were freeze-dried and kept at room temperature until needed for subsequent uses. The extraction yield ranged between 4 and 5%.

### 2.4. Total Phenolic and Flavonoid Content

The lyophilized extracts were redissolved in water (1 mg extract/mL), and total phenolic content (TPC) and total flavonoid content (TFC) were evaluated, respectively, using a modified Folin–Ciocalteu method and the aluminum chloride colorimetric methods, as described in detail by Moreira et al. [18].

### 2.5. Identification and Quantification of Phenolic Compounds Using HPLC-DAD Analysis

Lyophilized extracts were dissolved in water (Sample 1—20 mg/mL; Sample 2—22.5 mg/mL; Sample 3—29 mg/mL; Sample 4—22 mg/mL; Sample 5—29 mg/mL), and after filtration were analyzed in triplicate on an analytical HPLC-DAD unit (Shimadzu) using the gradient described by Mancini et al. [19]. Spectral data from all peaks were collected in the 200–500 nm range, and chromatograms were recorded at 280, 320, 340, and 350 nm. Data were processed on LabSolutions software. Compounds were identified by comparing their retention times and UV–vis spectra with standards injected under the same conditions.

External calibration curves (Table 1) were prepared to quantify the identified compounds. All of them were quantified with their corresponding standards, except for compounds 2, 3, 5, 6, 7, 11, 12, 16, 17 (quantified as p-coumaric acid), 19 (as 5-O-caffeoylquinic acid), 15 (as apigenin-6-C-glucoside), 21, 22, 29 (as kaempferol-3-O-glucoside), 26 (as quercetin-3-O-glucoside), and 30 and 31 (as tiliroside). The limits of detection (LOD) and quantification (LOQ) were calculated as LOD = 3.3 σ/S and LOQ = 10 σ/S, where σ and S were, respectively, the residual standard deviation and the slope of the curve.

### 2.6. Determination of Antioxidant Potential

#### 2.6.1. FRAP, DPPH^•^, and ABTS^•+^ Assays

The antioxidant activity of moringa extracts (1 mg extract/mL in water) was evaluated by three complementary in vitro assays, namely FRAP, DPPH^•^, and ABTS^•+^, according to the procedures reported by Moreira et al. [18] and Mendes et al. [20].

#### 2.6.2. Scavenging of Hydrogen Peroxide

Lyophilized extracts were dissolved in K_2_HPO_4_/KH_2_PO_4_ buffer (0.1 M, pH 7.4), and stock solutions with concentrations in the reaction volume of 0.19 (Samples 1 and 3), 0.16 (Sample 2), 0.23 (Sample 4), and 0.21 (Sample 5) mg/mL were prepared. From these stock solutions, serial dilutions were made to test 6 dilutions of each sample. H_2_O_2_ scavenging activity was evaluated according to a previously described methodology [19]. The absorbance at 230 nm was read with a spectrophotometer (Shimadzu UV-260 spectrophotometer, Kyoto, Japan) after 10 min incubation. Three assays were performed, each assay in triplicate (*n* = 3), and the results obtained were compared with Ascorbic acid (AA) (IC50 = 51.2 ± 4.6 µg/mL).

#### 2.6.3. Scavenging of Nitric Oxide

The method established by Soares et al. [21] was employed to ascertain the scavenging activity of ^•^NO. The extracts were prepared in K_2_HPO_4_/KH_2_PO_4_ buffer (0.1 M, pH 7.4), and stock solutions with concentrations in the reaction volume of 1.50 (Sample 1), 1.63 (Sample 2), 1.79 (Sample 3), 3.83 (Sample 4), and 3.50 (Sample 5) mg/mL were prepared. After that, serial dilutions were made to test 6 dilutions of each sample. AA was used as a positive control (IC50 = 470 ± 88 µg/mL). Three assays (*n* = 3) were performed, each in triplicate.

### 2.7. Cholinesterases Inhibition

Cholinesterases inhibitory activities were assessed using a 96-well microplate reader, according to Ellman’s previous method [21]. Extracts were dissolved in tris-HCl buffer (50 mM, pH 8). Stock solutions of each sample were prepared at final concentrations of 0.85 (Sample 1), 0.80 (Samples 2 and 5), 0.87 (Sample 3), and 0.86 (Sample 4). Serial dilutions were made from the stock solutions to test 6 dilutions of each sample. Three assays (*n* = 3) were performed, each one in triplicate, and the results compared with those obtained for galantamine (IC50 = 0.920 ± 0.131 µg/mL for AChE and IC50 = 4.92 ± 0.21 µg/mL for BuChE).

### 2.8. Quantification of Lead and Cadmium in Moringa Extracts

Pb and Cd were measured using a ContrAA 700 High-Resolution Continuum Source Flame Atomic Absorption Spectrometer from Analytik Jena (Jena, Germany). The measurements were taken at 283 nm for Pb and 228 nm for Cd, utilizing the Graphite Furnace module that included an MPE60 autosampler from Analytik Jena. The operational parameters were adjusted for the highest absorbance and the lowest background values, as shown in Table 2 and Table 3. Every day, external calibration curves were created using at least six standards. All extracts were twice prepared in water (1 mg/mL), filtered, and measurements taken, at minimum, in triplicate.

### 2.9. Consumer Perception

#### 2.9.1. Sensory Panel

The study underwent a review process and subsequently received approval from the Instituto Federal de Minas Gerais IRB, and before participation each individual gave their informed consent. The sensory panel comprised 82 consumers (56% female, 44% male, aged between 18 and 50).

#### 2.9.2. Sensory Analysis

Sensory assessments were executed in a regulated sensory atmosphere in compliance with best sensory practices [22]. The sensory panel tasters (56% women and 44% men, aged between 20 and 50) were tea consumers with a frequency of three times a week, and most of them bought their teas/herbal teas at the supermarket. The samples (25 mL) were provided in disposable cups, marked with a three-digit code, and offered in a balanced and randomized sequence as per the method of Wakeling and Macfie [23]. The beverages’ serving temperature was 60 ± 5 °C. Beverages were prepared throughout the sensory analysis period and were kept warm in closed thermos bottles (1 L).

The same panelists performed the Check All That Apply (CATA) method, acceptance, and tests to determine the intention to purchase. Tests for acceptance and intention to purchase were conducted both with and without providing information about the tea: “Moringa is considered a miracle tree of nature and a superfood. It presents a wide range of nutrients, including antioxidants, amino acids, vitamins, minerals, phytonutrients, and proteins, being, therefore, considered beneficial to health”. A focus group was utilized to generate CATA terms [24]. An experienced moderator, consistent across all meetings, conducted the sessions in a room spacious enough to host the participants, who were tea consumers, comfortably. The discussions encompassed sensory aspects of appearance, aroma, and flavor attributes. In the course of the test, consumers were requested to mark all CATA terms they deemed suitable for characterizing each beverage. They also assigned an acceptance grade to each sample using a hedonic scale that ranged from “extremely disliked” to “extremely liked”, and a grade for the purchase intention, utilizing a five-point scale that spanned from “I would definitely not buy” to “I would definitely buy” [25].

#### 2.9.3. Word Association and Label Information

The participants were asked to engage in a word association (WA) task, where they were encouraged to freely express the initial words, terms, sensations, associations, thoughts, or feelings that arose when consuming tea [26,27]. Moreover, they also pointed out the label information they paid attention to during their tea purchase. Following the completion of the WA task, participants were then asked to respond to a series of sociodemographic questions.

### 2.10. Statistical Analysis

IC50 values for bioactivities were calculated using GraphPad Prism 8 software (GraphPad Software, San Diego, CA, USA), and values were compared with one-way ANOVA using Tukey’s post-test. Results obtained with TPC, TFC and antioxidant assays were correlated with Pearson’s correlation coefficient, using the same software. The chemical data were analyzed using a one-way ANOVA with SPSS (IBM SPSS Statistics for Windows, Version 28.0. Armonk, NY, USA) statistical package, and the means were compared using the Tukey’s multiple range test (*p* < 0.05).

Sensory data were assessed using the Sensomaker [28], SPSS (IBM SPSS Statistics for Windows, Version 22.0 Armonk) and XLSTAT (Addinsoft, France, 2015) software. First, a preference mapping was obtained of moringa beverages’ sensory acceptance and CATA terms to clarify the sensory perceptions that most contributed to the sample’s acceptance [29], determining the drivers of liking moringa beverages. A principal components analysis (PCA) was performed to link the chemical and the sensory data to explore the influence of chemical composition on sensory perceptions [30]. To conduct the PCA, an m × n matrix was employed with m representing the number of samples and n denoting the number of variables [31]. The data underwent autoscaling, and the PCA routines were executed using the Chemoface software [29].

To assess the impact of information on the beverages purchase by male and female audiences, the product information (moringa tea benefits) effect on the purchase intention per gender was evaluated with a *t*-test for non-parametric samples.

To assess consumer perceptions through words and expressions that referred to the product, all associations made by the participants were selected, and the most recurring terms were selected. Then, terms were grouped by triangulation, and synonyms mentioned by more than 5% of the consumers were grouped in the same category [32]. Based on previous studies, this selected threshold helped to prevent significant information loss [33,34,35]. The Global Chi-square test was used to assess differences in the frequency of the mentioned terms between consumers’ moringa tea groups. Subsequently, after noting the apparent discrepancies, the Chi-square per cell test was utilized to identify the variation source of the Global Chi-square [36,37,38].

A word cloud was also obtained from the words, phrases, sensations, connections, notions, or emotions that were evoked when moringa beverage consumption was introduced for (A) all the tasters, (B) male tasters, and (C) female tasters for better visualization.

## 3. Results and Discussion

### 3.1. Total Phenolic and Flavonoid Content

**Figure 1:**
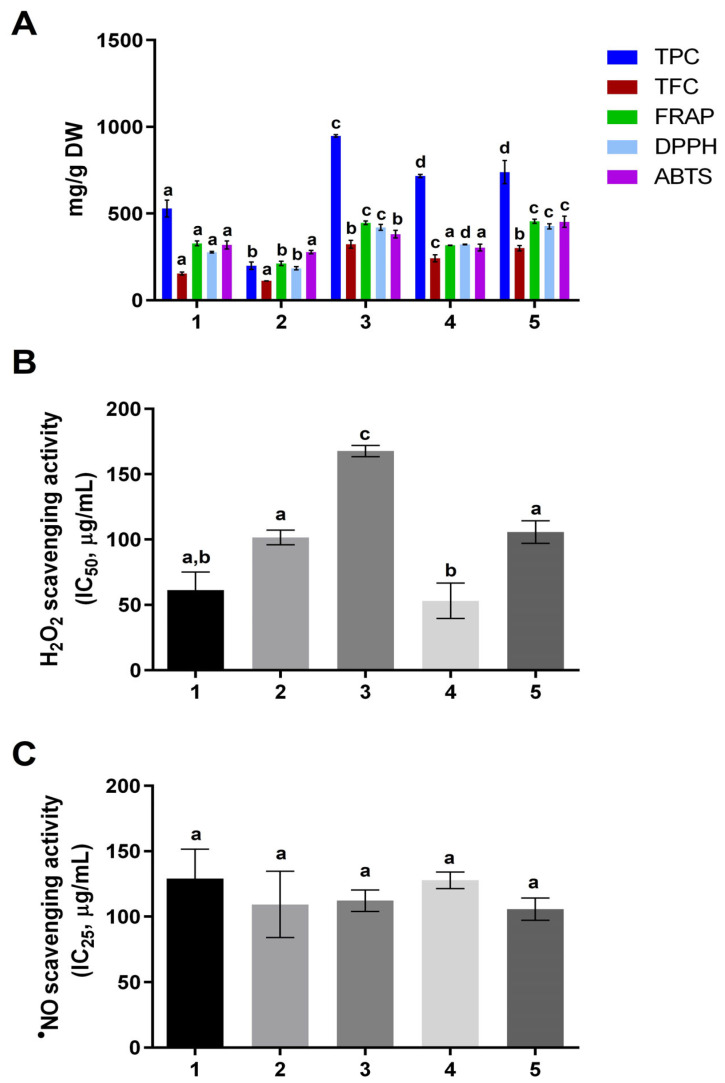
Total phenolic composition and antioxidant activities of the samples. (**A**) TPC (mg GAE/g dw) and TFC (mg EE/g dw) and antioxidant activity of *M. oleifera* tea extracts obtained with FRAP (mg AAE/g dw), DPPH^•^ (mg TE/g dw), and ABTS^•+^ (mg TE/g dw) assays; (**B**) H_2_O_2_ scavenging activity and (**C**) ^•^NO scavenging activity. Within the bar for each analysis, homogenous groups are shown by the same letter (ANOVA followed by Tukey’s test) for *p* < 0.05.

The content of phenolic or flavonoid compounds could be correlated with antioxidant activity, reflecting the sample’s potential to act as an inherent provider of antioxidants. These results are shown in Figure 1.

Figure 1A represents the obtained values for TPC and TFC assays for the *M. oleifera* samples analyzed.

Concerning TPC (Figure 1A), the extracts from Sample 3 (cold drink) exhibited the highest quantity of phenolic compounds (948 ± 7 mg GAE/g dw), followed by Samples 4 and 5, then Sample 1. At the same time, a significantly lower level was determined in Sample 2 (200 ± 21 mg GAE/g dw). Significant differences existed between the analyzed samples, except for 4 and 5 (*p* > 0.05). The obtained results demonstrated that the sample type influences the content of phenolic compounds recovered from moringa. Comparing our TPC values with available information in the literature, it was observed that results from the present study were at the same level as the values reported by Ilesanmi et al. [39] for 80% aqueous methanolic extracts of *M. oleifera* leaves (541 ± 1 mg GAE/g dw), but higher than those reported by other researchers [40,41,42,43,44]. Indeed, our TPC findings were higher than those of *M. oleifera* trees found in the Menzel Lahbib (Gabes-Tunisia) region, whose values ranged from 80.9 ± 1.2 to 136 ± 8 mg GAE/g dw. Nwidu et al. [43] also evaluated the phenolic content from methanolic, aqueous, and ethanolic extracts of different parts (namely leaf, bark, root, seed, and flower) from *M. oleifera* from two locations in the Niger Delta. The TPC values reported were also lower than those obtained in the present study (values ranging from 94.5 ± 0.9 to 287 ± 1 mg GAE/g dw), demonstrating that the sample’s geographical origin influences the number of phenolic compounds. In addition, these authors also reported different TPC levels depending on the solvent employed and the moringa part used to recover the phenolic compounds. They concluded that root extracts presented the highest TPC (287 ± 1 mg GAE/g dw for methanolic extract), while the ethanolic and methanolic leaf extracts presented lower levels (201 ± 2 and 113 ± 2 mg GAE/g dw, respectively). In another study, Rocchetti et al. [44] also tested similar extraction techniques, namely maceration, homogenizer-assisted extraction, rapid solid–liquid dynamic extraction, MAE and UAE, and the solvent effect (methanol 100% and methanol/water 50:50, *v*/*v*), and also concluded that both factors influenced the polyphenols’ recovery from *M. oleifera* leaves. These authors reported that the homogenizer-assisted extraction of *M. oleifera* leaves with 100% methanol extracted the highest polyphenols (35.2 mg GAE/g dw) [44]. These findings demonstrated that the extraction technique, solvent choice, and geographical origin of *M. oleifera* leaves substantially impacted the recovery of polyphenols.

The same trend as previously reported for TPC was observed regarding the TFC of *M. oleifera* extracts, also summarized in Figure 1A. The highest TFC was observed for extracts from Sample 3 (323 ± 23 mg EE/g dw), followed by Sample 5 > Sample 4 > Sample 1 > Sample 2. There were, however, no significant differences (*p* > 0.05) between Samples 3 and 5 and between Samples 1 and 2. Unfortunately, a direct comparison of the obtained flavonoid content with data from the literature cannot be performed, as most of the studies employed other standards (such as rutin or quercetin) to express the TFC values [40,41,42,43]. However, a good correlation coefficient between the TPC and TFC assays was observed (r = 0.887, data not shown), which agreed with that previously reported by other authors for *M. oleifera* leaves [42,43], revealing that Sample 3 could be the most potent natural source of bioactive compounds.

### 3.2. Phenolic Composition of M. oleifera Extracts

**Figure 2:**
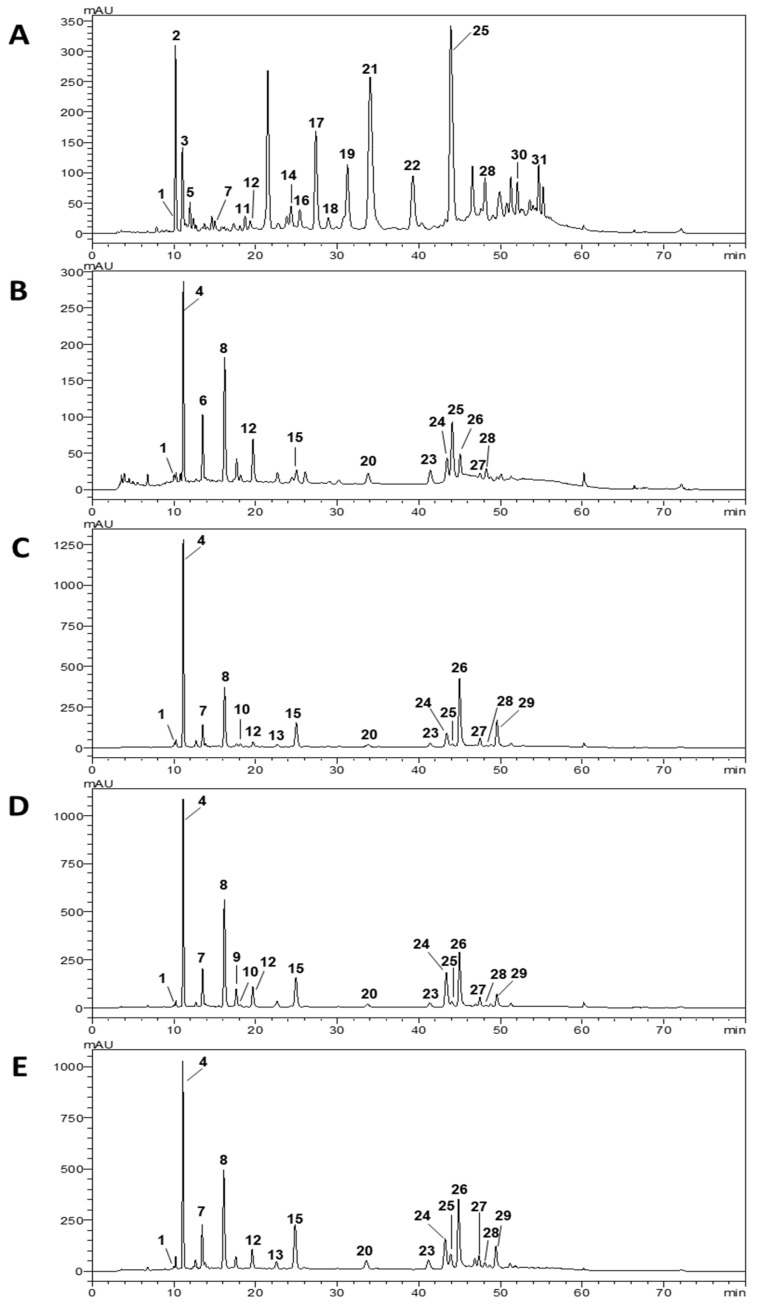
HPLC-DAD chromatograms (320 nm) of *M. oleifera*. (**A**)—Sample 1; (**B**)—Sample 2; (**C**)—Sample 3; (**D**)—Sample 4; (**E**)—Sample 5. Identity of the peaks as in Table 4.

In the five extracts prepared from *M. oleifera* leaves, HPLC-DAD analysis identified thirty-one phenolic compounds (Figure 2, Table 4), which included protocatechuic acid (**1**), derivatives of p-coumaric acid and p-coumaroylquinic acid (**2**, **3**, **5**–**7**, **11**, **12**, **14**, **16**, and **17**), caffeic acid and caffeoylquinic acids (**4**, **8**, **9**, and **10**), ferulic acid and feruloylquinic acid (**13** and **18**), apigenin-C- and di-C-glycosides (**15**, **20**, and **23**), kaempferol-3-O-glycosides and other kaempferol derivatives (**21**, **22**, **27**–**29**), and quercetin-3-O-glycosides and a quercetin derivative (**24–26**), as well as two acylated kaempferol derivatives (**30** and **31**). The total phenolic content obtained from the HPLC analysis was in the order of Sample 2 (14.2 mg/g) < Sample 3 (30.5 mg/g) < Sample 5 (35.8 mg/g) < Sample 4 (37.9 mg/g) < Sample 1 (59.6 mg/g). Among all samples, Sample 1 stands out for its chemical profile, significantly different from the other four samples (*p* < 0.05), characterized by the predominance of *p*-coumaroylquinic acid derivatives, the majority only present in this sample, high levels of quercetin-3-*O*-rutinoside (**25**) with 21.0 ± 0.1 mg/g of extract, and an absence of quercetin-3-*O*-glucosidase (**24**). On the other hand, Samples 2 to 5 displayed similar phenolic profiles, with 3-*O*-caffeoylquinic acid (**4**), 4-*O*-caffeoylquinic acid (**8**), apigenin-6,8-di-*C*-glucoside (**15**), and a quercetin derivative (**26**) as their main compounds. Moreover, while quercetin-3-*O*-glucoside (**24**) content was higher than that of quercetin-3-*O*-rutinoside (**25**) in Samples 3, 4, and 5, the opposite was observed for Sample 2. Sample 2 contained the lowest amount of identified phenolic compounds, which agreed with the results obtained for the TPC and TFC assays. On the other hand, Sample 3 presented the highest TPC and TFC levels, followed by Samples 4 and 5. However, a low correlation was found between HPLC quantifications and TPC (0.0855) and TFC (0.00190) values. In spectrophotometric methods such as TPC and TFC, non-phenolic soluble compounds such as sugars and proteins could act as interfering compounds that overestimate the overall content. The results obtained from the chromatographic method were usually considered interference-free [45]. Moringa leaves have been reported to present a soluble sugar content of 12.0 ± 0.7 mg/g dw [46] and a total protein content of up to 30% [47]. Sample 3 was presented as highly soluble powdered moringa leaves, which could increase the extraction of interfering compounds compared with whole or crushed dry leaves, explaining the higher TPC and TFC values.

As pointed out for TPC values, data in the literature have shown the significant influence of the geographical origin on the chemical composition reported for samples collected in Africa [41,42,48,49], Asia [50,51,52,53], America [54], and Europe [44], with authors reporting different glycoside derivatives of flavonols and flavones, the presence/absence of hydroxycinnamic and hydroxybenzoic acids, and the presence/absence of anthocyanins, isoflavones, and chalcones, among other compounds. Nevertheless, all compounds detected in the studied samples have already been found in aqueous, hydroalcoholic, methanolic, and acetonic extracts of *M. oleifera* leaves collected in the regions mentioned above.

Indeed, it is well-described that *M. oleifera* ecotypes are phenotypically different and possess distinct secondary metabolite profiles [55]. Makita et al. [48] investigated the distribution patterns of three flavonol rutinosides in twelve cultivars of *M. oleifera* from Thailand, Taiwan, the USA, and South Africa. Different compositions were found, reflecting the influence of the environmental conditions and genetic pool. Similarly, Förster et al. [55] showed morphological and chemical differences among six ecotypes of *M. oleifera* from the USA, Thailand, India, the Philippines, and Taiwan.

### 3.3. Antioxidant Potential

#### 3.3.1. FRAP, DPPH^•^, and ABTS^•+^ Assays

The findings related to the antioxidant activity of *M. oleifera* extracts are depicted in Figure 1A.

The FRAP assay used to evaluate antioxidant activity indicated that Sample 5 (455 ± 12 mg Ascorbic Acid Equivalents (AAE)/g dw) demonstrated the most potent ferric reducing ability, similar to that reported for Sample 3 (446 ± 11 mg AAE/g dw), with no significant differences between these two samples (*p* > 0.05). Sample 2, on the other hand, led to the lowest antioxidant activity determined by the FRAP assay (212 ± 13 mg AAE/g dw), which followed the lowest TPC and TFC values (*p* < 0.05).

DPPH^•^ radical scavenging activity agreed with ferric-reducing values. Samples 3 and 5 exhibited the greatest scavenging activity against DPPH^•^ radicals (*p* > 0.05) (420 ± 17 and 426 ± 14 mg Trolox Equivalents (TE)/g dw, respectively), followed by Sample 4, Sample 1, and finally Sample 2 (322 ± 1, 277 ± 5 and 185 ± 10 mg TE/g dw, respectively). As aforementioned, the observed differences could be attributed not only to the moringa extracts’ geographical origin, but also to their chemical composition and their phenolic and flavonoid contents, as antioxidant activity is strongly related to these parameters. The outcomes acquired in this study highlighted that flavonoid compounds are key contributors to the antioxidant attributes of *M. oleifera* extract, as they demonstrate a higher correlation with TFC (r = 0.844 for FRAP and r = 0.947 for DPPH^•^) than with TPC (r = 0.787 for FRAP and r = 0.864 for DPPH^•^).

A slightly different trend was observed concerning the ABTS^•+^ assay, and a less significant correlation was observed with TPC (r = 0.418) and TFC (r = 0.626). However, in line with previous results, Sample 5 displayed the higher ABTS^•+^ radical scavenging activity (468 ± 23 mg TE/g dw), followed by Sample 3 (393 ± 13 mg TE/g dw), while Sample 2 exhibited the lowest activity (281 ± 12 mg TE/g dw). There were no significant differences between Samples 1, 2, and 4 for this radical.

Regarding comparing obtained data in the present study with available information in the literature, it was only possible to compare our results from the DPPH^•^ assay with the values reported by Braham et al. [42]. These authors reported the maximum DPPH^•^ radical scavenging activity for *M. oleifera* leaf extracts prepared using acetone 70% (183 mg TE/g dw), which was twofold lower than the maximum activity observed for Sample 5.

The results confirmed that *M. oleifera* samples represent a source of powerful antioxidants, with Sample 3 presenting the highest TPC and TFC values. In contrast, the highest antioxidant activity was exhibited by Sample 5. According to the HPLC analysis (Table 4), Samples 5 and 3 presented a higher flavonoid content, particularly apigenin and kaempferol derivatives, respectively. Flavonoids are known to directly scavenge oxygen or nitrogen radical species through hydrogen atom donation, double bonds in the phenolic ring, hydroxyl side chains, and the glycosylation of anthocyanidins [56]. The variation in the reported results could be explained by the diverse geographical locations and the variation in the climatic and edaphic factors [41]. Other aspects, such as the origin of the raw materials, the timing of collection, the drying method used, and the extraction technique, will also influence the moringa samples’ chemical composition, phenolic compounds, and biological activities [41].

#### 3.3.2. ROS and RNS Scavenging Activity

Strong activity was observed for the aqueous extracts studied against H_2_O_2_, with values of IC_50_ ranging from 53.1 ± 13.5 µg/mL (Sample 4) to 168 ± 4 µg/mL (Sample 3). Extracts 1 and 4 were found to be as active as AA (IC_50_ = 51.2 ± 4.6 µg/mL), showing stronger activity than Samples 2, 3, and 5 (*p* < 0.05) (Figure 1B). As far as we know, only methanolic and hydroalcoholic extracts from the leaves have been tested against H_2_O_2_. An 80% methanol extract from the leaves of *M. oleifera* displayed better activity against H_2_O_2_ than the positive control (AA) [57]. However, in another study, methanolic extracts prepared from leaves, stems, and stalks were reported as less active than AA [58]. Karthivashan et al. [50] described hydroethanolic extracts’ strong H_2_O_2_ scavenging activity from *M. oleifera* leaves.

The aqueous extracts prepared from the five samples of *M. oleifera* showed moderate activity against ^•^NO, displaying IC_25_ values between 129 ± 22 µg/mL (Sample 1) and 106 ± 9 µg/mL (Sample 5) (*p* > 0.05) (Figure 1C). To the best of our knowledge, only one previous study reported the ^•^NO scavenging activity of aqueous extracts from *M. oleifera* leaves [59]. The extract from mature leaves was slightly more active than that from tender ones (IC50 = 56.8 µg/mL and 65.9 µg/mL, respectively), showing a positive correlation between TPC and antioxidant activity. Comparing this result with those obtained in the current study, in our case it was not possible to reach 50% of inhibition with the extracts prepared.

Moreover, other parts of *M. oleifera* have been tested against this RNS. Masum et al. [60] evaluated the ^•^NO scavenging activity of the leaf, bark, and fruit of *M. oleifera*. Ethyl acetate and chloroform fractions were the most active ones, containing the highest TPC but not the highest TFC. An 80% methanolic extract prepared with the leaves was also reported by Ayoola et al. [40]. However, the extract did not scavenge the radical in a dose–response manner, displaying % inhibition in the range of 59.5% (at 6.25 µg/mL) and 50.4% (at 100 µg/mL). *M. oleifera* seeds were also active ^•^NO scavengers. Jahan et al. [61] tested the aqueous, methanolic, and acetone extracts produced with the seeds and noted a positive correlation between the scavenging activity and both TPC and TFC, yet a negative correlation with the content of tannins. The aqueous extract was the most active (IC_50_ = 218 ± 1 μg/mL).

### 3.4. Cholinesterase Inhibition

To our knowledge, the aqueous extract obtained from leaves of *M. oleifera* was never tested against AChE. The aqueous extracts displayed weak activity against both enzymes in the current study. At the highest tested concentration (~0.8 mg/mL), the following % inhibitions were obtained for AChE: 27.7 ± 11.2 (Sample 1), 32.4 ± 3.4 (Sample 2), 32.0 ± 6.4 (Sample 3), 17.6 ± 9.3 (Sample 4), and 15.6 ± 1.2 (Sample 5).

Concerning other extraction solvents, Nwidu et al. [43] tested methanolic, ethanolic, and aqueous extracts prepared from different parts of *M. oleifera* against mammalian AChE. However, the aqueous leaf extract was not evaluated in the bioassay. Among 19 extracts tested, they noticed that the AChE inhibitory strength of different parts of the plant followed this order: root > bark > leaf > flowers > seed. The lowest IC50 values were obtained for some methanolic and ethanolic extracts. Furthermore, an inverse relationship between the AChE inhibition and TPC and TFC of the extracts was also shown, which agrees with the results reported herein since a low correlation was found between AChE vs. TPC (r = 0.103) and AChE vs. TFC (r = 0.162).

Moreover, the AChE inhibitory activity of a methanolic extract obtained from the leaves has been tested by other authors. In vitro and in vivo assays resulted in 29% and 34% inhibition of zebrafish AChE, respectively [62]. Rocchetti et al. [44] also reported that the methanolic and ethyl acetate extracts from the leaves displayed some degree of AChE and BuChE inhibition, which was not correlated with the phytochemicals identified, namely, anthocyanins, flavonols, flavones, lignans, tyrosols, alkylphenols, phenolic acids, and stilbenes.

### 3.5. Quantification of Pb and Cd in Moringa Extracts

The quantification of Pb and Cd in aqueous moringa extracts is presented in Table 5.

The highest Pb content was found in Sample 2, with 1.46 ± 0.12 mg/kg dw, followed by Sample 5 with 1.35 ± 0.09 mg/kg dw. The sample with the lowest Pb value was Sample 1, which presented 0.108 ± 0.011 mg/kg dw. Pb values differed significantly, except for Samples 3 and 4 (*p* > 0.05). Regarding Cd, the sample that contained the highest concentration was Sample 3, which presented 0.702 ± 0.031 mg/kg dw, followed by Sample 4 with 0.275 ± 0.035 mg/kg dw (*p* < 0.05). Pb and Cd are toxic, especially for the kidney and nervous system [9]. The highest acceptable concentrations recommended by WHO for Cd and Pb are 0.3 and 10 mg/kg, respectively, in all plant parts of herbal products for human consumption [63]. Regarding Pb, all the reported values were below the maximum limit (10 mg/kg). However, Sample 3 presented a higher Cd content (0.702 ± 0.031 mg/kg dw) than the recommended value of 0.3 mg/kg. Tea-like beverages are essentially colloidal solutions. The levels of contaminants, such as heavy metals, within these solutions are predominantly determined by factors such as infusion time, temperature, and the chemical composition of the leaves. These contaminants can exist in both the solution and the colloidal material [64]. In the context of this study, only the solution was subject to analysis. Various studies have indicated a strong correlation between the proportion of elements leached into the infusion and the beverage’s tannin content; lower tannin levels often lead to improved leaching. Although this study did not assess the tannin content of moringa leaves, reported values typically range from 6.84 ± 0.05 to 10.22 ± 1.11 mg GAE/100 g in dry moringa leaves [65]. These figures are relatively high when compared to other beverages such as hibiscus, mate, green, and black teas, which have tannin content percentages of 0.3%, 4.0%, 5.4%, and 6.5%, respectively [66].

Biel et al. [67] reported a mean Pb concentration of 2.09 mg/kg in the leaves of *M. oleifera* obtained from India, but the authors did not find Cd in their extracts. Aissi et al. [68] evaluated Pb, and Cd contamination in *M. oleifera* leaf powders obtained in the Cotonou market (Benin) and reported mean values of 1.526 and 0.246 mg/kg for Pb and Cd, respectively. Therefore, the results reported in Table 5 are comparable with the literature. Environmental hazards related to heavy metals depend on their presence and the soil’s ability to absorb them. About half of the global contamination sites are due to heavy metals, a statistic more prevalent in developed nations because of their industrial use of these elements [7]. Geographically, north-eastern and eastern-central Europe suffer less from heavy metal contamination, while western Europe and the Mediterranean often exceed thresholds. Outside of Europe, Africa’s less developed nations face heavy metal contamination primarily due to inadequate regulation and waste management. Notably, Cd and Pb are widespread across the continent. China, the largest Asian country, grapples with significant heavy metal pollution due to rapid development. Over a quarter of China’s arable land is contaminated with heavy metals, with higher concentrations observed in regions rich in mineral resources [7]. Given the limited sample size analyzed in this study, establishing a correlation between the origin of the leaves and the extent of heavy metal contamination was impossible.

### 3.6. Consumer Perceptions

**Figure 3:**
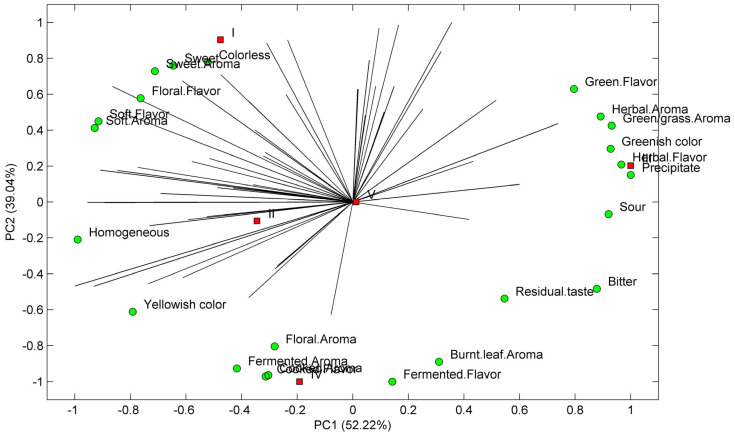
Preference mapping obtained from CATA terms and moringa beverages’ sensory acceptance. *M. oleifera* beverages Sample 1, Sample 2, Sample 3, Sample 4, and Sample 5 are denoted as follows: I, II, III, IV, and V, respectively.

Preference mapping (Figure 3) illustrates the sensory attributes contributing significantly to beverage acceptance. From the panelists’ list of terms encompassing sensory modalities of appearance, aroma, and flavor attributes, sweet taste, aroma, and floral flavor were identified as factors driving the liking of moringa tea. Conversely, green, grass, and herbal flavors, along with sour, bitter, and the presence of precipitate, were considered unfavorable sensory attributes.

**Figure 4:**
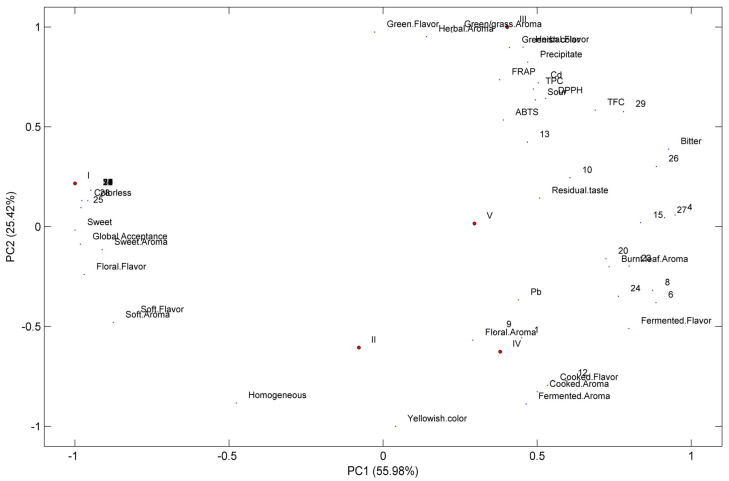
Principal component analysis applied to the quality indexes (Cd; Pb; TPC; TFC; FRAP; DPPH; ABTS), phenolic composition (compounds **1**–**31** named in Table 4), sensory attributes obtained from CATA test, and global sensory acceptance from moringa beverages. The beverages of *M. oleifera* are labeled I, II, II, IV, and V, corresponding to commercial Sample 1, Sample 2, Sample 3, Sample 4, and Sample 5, respectively.

Zhang et al. [69] also noted that consumers prefer sweet and umami tastes while generally disliking bitter and astringent tastes. This consumer acceptance can be explained by the composition of each analyzed sample, as depicted in Figure 4.

Phenolic content and composition seem to influence consumers’ tastes and preferences. Sample 1 was the preferred sample. It was characterized by a sweet, floral, soft flavor and the presence of quercetin-3-O-rutinoside (**25**). Being homogeneous and colorless also contributed to a better acceptance of moringa beverages 1 and 2. Conversely, Samples 3, 4, and 5, which presented higher antioxidant values, were less preferred. Sample 3 was related to green and herbal flavors, the beverages’ TPC, TFC and antioxidant activities, and the presence of 3-O-feruloylquinic acid (**13**) and a kaempferol derivative (**29**). The floral aroma characterized Tea IV with the presence of protocatechuic acid (**1**) and 5-O-caffeoylquinic acid (**9**), while a cooked flavor and cooked and fermented aroma were related to a p-coumaroylquinic acid derivative (**12**). A residual flavor was related to caffeic acid (**10**), while a bitter taste was linked to a quercetin derivative (**26**). Sample 1 (the most preferred) had grades between 6 (I liked it slightly) and 7 (I liked it moderately), followed by Sample 2. Sample 1 presented a phenolic profile different from the other samples analyzed; it was mainly composed of *p*-coumaroylquinic acid derivatives (Figure 2, Table 4), which were practically absent in the other four samples, which can explain the different consumer preferences [69,70]. Sample 2, despite the low TPC, TFC, and antioxidant activity, was also one of the favorite tea samples. The opposite behavior was also observed for Sample 3, which was very promising from the antioxidant and chemical point of view; however, it was the least preferred one.

According to Zhang et al. [69], flavonoid glycosides are astringent compounds, while caffeoylquinic acid derivatives are bitter. Since the most preferred beverage was Sample 1 (containing a high amount of astringent quercetin-3-O-rutinoside), we cannot neglect the possible contribution of other non-quantified compounds, such as amino acids, that could contribute to the sweetness of Sample 1 [69]. Regarding Samples 2, 3, 4, and 5, the different caffeoylquinic acid derivatives identified (Section 3.2) could also be responsible for the bad sensory perception reported by the tasters [69].

To understand the influence of the information regarding the health benefits of the beverages on consumer’ preferences, two series of sensory tests were performed with moringa beverages. One, in which tasters were not provided with information about moringa beverages’ health properties, and the other, in which a short text explaining the benefits of drinking these beverages was given before the sensory testing. It was noted that when the claim of moringa beverages’ benefits was presented, a greater acceptance of Sample 5 (*p* = 0.025) was noticed. Moreover, the information effect was more relevant to leveraging the purchasing intention of women (*p* < 0.05). According to the results obtained through the global chi-square analysis (*p* < 0.05), there were significant differences in the perception of the two groups of tea consumers (women and men). Table 6 shows the terms associated with tea consumption and reading labels according to the consumers’ perceptions. The most observed label information during tea purchase were health benefits, followed by ingredients and type/flavor, particularly by women. However, 25% of consumers said they ignore this information when buying tea. Utilizing the chi-square per cell tool, we found that among the obtained frequencies, the term “I do not notice” was significantly more frequently chosen (*p* < 0.05) by the male respondents than expected. This reaffirms the importance of the awareness of reading labels and checking the origin of the products and the presence of contaminants.

Moreover, encouraging the recognition by the consumer of health benefits promoted by the product adds value to the product. De Godoy et al. [71] reported that the purchase of mate tea was greatly affected by product quality, brand, and price. They also indicated that most consumers showed dissatisfaction with the products available on the market due to their low quality and packaging [71]. These aspects reinforce the need to improve tea labeling.

Panelists were also prompted to spontaneously complete a WA task. In this task, they were asked to share the initial words, expressions, sensations, associations, thoughts, or feelings that came to mind upon tasting the moringa beverages. Figure 3 shows the preference mapping obtained from moringa beverages’ sensory acceptance and CATA terms.

**Figure 5:**
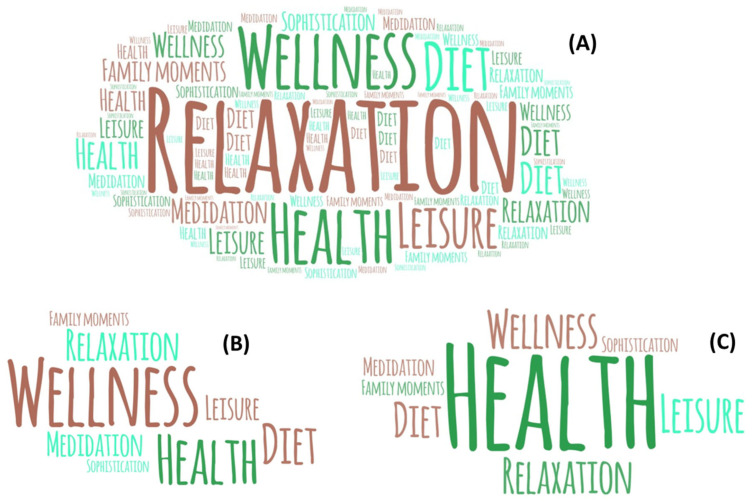
Word cloud obtained from first words, terms, sensations, associations, thoughts, or feelings that came to mind when moringa beverage consumption was introduced for (**A**) all the tasters; (**B**) male tasters; and (**C**) female tasters.

Health, wellness, relaxation, and leisure were among the most common terms/feelings associated with moringa beverages consumed by consumers, as seen in Table 6 and Figure 5. Health presented a significantly higher value (*p* < 0.05) than expected in the female public. In comparison, male consumers’ wellness values presented a higher value (*p* < 0.05) (Table 6 and Figure 5). These aspects can also be explored in tea labeling. Indeed, some authors have already highlighted the same findings. Swahn et al. [72] reported a shopping habits study showing that products describing their sensory features can influence consumers’ choices. Another study by Mueller et al. [73] noted that wine bottles with labels presenting different information, including sensory descriptions, guided consumer decision-making positively.

In the present study, as taste is demonstrated to be among the most relevant information, including the product’s sensory profile as a ‘sensory claim’ on the label seemed interesting and potentially explorable. ‘Sensory claims’ pertain to sensory experiences and product traits concerning factors such as taste, texture, visual appeal, smell, or auditory cues. They can be described as “with fruity and healthy taste” or “with floral and relaxing aroma”, used by producers to promote a product [74]. These “sensory claims” enable market participants to promote a product positively and strategically position it in the market [74]. Therefore, the drivers of liking determined in this study for moringa beverages with words/feelings association represent critical information for tea labeling to highlight health benefits. Therefore, prospective consumers’ preferences and expectations, demanding new flavors, satisfaction and enjoyment for new food products, should guide food creators. Product diversity for gender audiences would also be interesting.

## 4. Conclusions

This study comprehensively assessed consumer perceptions and the acceptance of beverages derived from *M. oleifera*. This was achieved through a multidisciplinary approach, which involved scrutinizing these beverages’ sensory attributes, chemical composition, and bioactivity. These findings serve as valuable information for manufacturers to enhance their product labeling, formulate effective marketing strategies, and augment the overall value within the production chain. The importance of the strategic methodologies in this study was emphasized through a detailed understanding. The evaluation of sensory attributes is crucial, as it correlates with consumer preferences. By examining factors such as taste, texture, aroma, and appearance, one can decipher the sensory qualities most appealing to consumers, thereby directing manufacturers in creating beverages that are likely to be embraced by the market. Simultaneously, the analysis of the chemical composition of the beverages provides insights into the products’ nutritional properties and potential toxicity associated with the plant itself. By associating nutrient contents with health benefits, consumers can be educated about the potential health impacts of the beverages. This could enhance consumer trust and render the product more appealing to health-conscious individuals. Lastly, examining the bioactivity of *M. oleifera* beverages is imperative for substantiating the health claims associated with the product. By scientifically endorsing these claims, credibility is added to the product, allowing manufacturers to ground their marketing strategies in highlighting these health benefits. This comprehensive approach can be an effective instrument for understanding consumer preferences, guiding product development, and cultivating effective marketing strategies, ultimately contributing significant value to the entire production chain of *M. oleifera* beverages.

From the analyzed samples, the consumers’ most preferred sample (number 1) showed a phenolic profile different from the other four samples, mainly composed of p-coumaroylquinic acid derivatives and high levels of quercetin-3-O-rutinoside. In contrast, the others contained several caffeoylquinic derivatives and flavonol glycosides. On the other hand, Sample 3 was least appreciated by consumers; however, it displayed the highest TPC and TFC value and antioxidant activities: 948 ± 7 mg GAE/g dw, 323 ± 23 mg EE/g dw, and 420 ± 17 mg Trolox Equivalents (TE)/g dw, respectively. Regarding the Pb and Cd analysis, all the herbal products had concentrations lower than the recommended maximum intake based on current knowledge, except for Sample 3, which presented higher Cd levels (0.702 ± 0.031 mg/kg dw) than the acceptable value from WHO (0.3 mg/kg dw), reinforcing the need to provide appropriate guidelines and distribute them to medicinal plant producers and suppliers. To conclude, the generated results in the present study will contribute to gathering more information about this plant species, helping regulatory authorities to make the right decisions about medicinal plant commercialization.

## Figures and Tables

**Table 1:** Regression equations, *R*^2^, limits of quantification (LOQ) and limits of detection (LOD) of the HPLC standards.

Retention Time (min)	Compound	Regression Equation	*R* ^2^	LOD (mg/mL)	LOQ (mg/mL)
10.05	Protocatechuic acid	y = 3.25 × 10^7^*x* + 1.50 × 10^4^	0.9988	5.89 × 10^−4^	1.79 × 10^−3^
11.13	3-O-Caffeoylquinic acid	y = 7.29 × 10^7^*x* − 9.60 × 10^5^	0.9963	2.35 × 10^−5^	7.12 × 10^−5^
16.17	4-*O*-Caffeoylquinic acid	y = 6.81 × 10^7^*x* − 2.64 × 10^5^	0.9995	2.02 × 10^−6^	6.14 × 10^−6^
17.62	5-*O*-Caffeoylquinic acid	y = 8.11 × 10^7^*x* − 4.27 × 10^4^	0.9998	1.16 × 10^−4^	3.53 × 10^−4^
18.11	Caffeic acid	y = 1.34 × 10^8^*x* − 9.92 × 10^4^	0.9997	6.45 × 10^−6^	1.95 × 10^−5^
24.37	*p*-Coumaric acid	y = 1.43 × 10^8^*x* + 3.01 × 10^4^	0.9999	5.21 × 10^−6^	1.58 × 10^−5^
28.94	Ferulic acid	y = 1.27 × 10^8^*x* − 2.00 × 10^5^	0.9997	1.77 × 10^−5^	5.38 × 10^−5^
33.69	Apigenin-8-*C*-glucoside	y = 4.60 × 10^7^*x* − 8.32 × 10^3^	0.9998	4.56 × 10^−5^	1.38 × 10^−4^
41.30	Apigenin-6-*C*-glucoside	y = 6.69 × 10^7^*x* − 5.55 × 10^4^	0.9998	1.80 × 10^−5^	5.44 × 10^−5^
43.34	Quercetin-3-*O*-glucoside	y = 4.99 × 10^7^*x* + 1.14 × 10^4^	0.9999	2.92 × 10^−5^	8.86 × 10^−5^
44.00	Quercetin-3-*O*-rutinoside	y = 3.51 × 10^7^*x* + 3.22 × 10^4^	0.9992	1.91 × 10^−5^	5.79 × 10^−5^
47.41	Kaempferol-3-*O*-glucoside	y = 2.78 × 10^7^*x* − 2.11 × 10^4^	0.9998	1.20 × 10^−5^	3.64 × 10^−5^
48.17	Kaempferol-3-*O*-rutinoside	y = 3.56 × 10^7^*x +* 7.66 × 10^3^	0.9998	1.85 × 10^−5^	5.60 × 10^−5^
55.70	Tiliroside	y = 7.58 × 10^7^*x* − 2.87 × 10^4^	0.9992	3.05 × 10^−5^	9.26 × 10^−5^

**Table 2:** Optimized operational parameters for the graphite furnace analysis of Pb.

Step	Name	Temp. (°C)	Ramp (°C/s)	Hold (s)	Time (s)
1	Drying	80	6	20	29.2
2	Drying	90	3	20	23.3
3	Drying	110	5	10	14.0
4	Pyrolysis	350	50	20	24.8
5	Pyrolysis	800	300	10	11.5
6	Gas Adaptation	800	0	5	5.0
7	Atomize	1500	1500	4	4.5
8	Clean	2450	500	4	5.9

**Table 3:** Optimized operational parameters for the graphite furnace analysis of Cd.

Step	Name	Temp. (°C)	Ramp (°C/s)	Hold (s)	Time (s)
1	Drying	80	6	20	29.2
2	Drying	90	3	20	23.3
3	Drying	110	5	10	14.0
4	Pyrolysis	350	50	20	24.8
5	Pyrolysis	600	300	10	10.8
6	Gas Adaptation	600	0	5	5.0
7	Atomize	1200	1400	3	3.4
8	Clean	2450	500	4	6.5

**Table 4:** Phenolic composition (mg/g dried extract) of the *M. oleifera* samples.

Peak	Compounds	RT (min)	λmax (nm)	Sample 1 *	Sample 2 *	Sample 3 *	Sample 4 *	Sample 5 *
1	Protocatechuic acid	10.05	260, 294	0.276 ± 0.055 ^a^	0.827 ± 0.047 ^b^	0.402 ± 0.002 ^c^	0.523 ± 0.003 ^d^	0.786 ± 0.012 ^b^
2	*p*-Coumaroylquinic acid derivative 1	10.20	313	1.05 ± <0.01	-	-	-	-
3	*p*-Coumaroylquinic acid derivative 2	11.06	312	0.616 ± <0.001	-	-	-	-
4	3-*O*-Caffeoylquinic acid	11.13	248, 299 sh, 325	-	2.33 ± 0.01 ^a^	6.12 ± 0.02 ^b^	6.93 ± 0.01 ^c^	5.06 ± 0.03 ^d^
5	*p*-Coumaroylquinic acid derivative 3	11.98	312	0.236 ± <0.001	-	-	-	-
6	*p*-Coumaroylquinic acid derivative 4	13.50	310	-	0.391 ± 0.006 ^a^	0.410 ± 0.002 ^a^	0.775 ± <0.001 ^b^	0.665 ± 0.017 ^c^
7	*p*-Coumaroylquinic acid derivative 5	15.05	311	0.0990 ± <0.001	-	-	-	-
8	4-*O*-Caffeoylquinic acid	16.17	250, 299 sh, 326	-	2.03 ± 0.04 ^a^	2.88 ± 0.03 ^b^	5.52 ± <0.01 ^c^	3.93 ± 0.10 ^d^
9	5-*O*-Caffeoylquinic acid	17.62	252, 299 sh, 325	-	-	-	0.870 ± 0.011	-
10	Caffeic acid	18.11	250, 290 sh, 323	-	-	0.136 ± 0.003 ^a^	0.139 ± <0.001 ^a^	-
11	*p*-Coumaroylquinic acid derivative 6	18.72	312	0.132 ± <0.001	-	-	-	-
12	*p*-Coumaroylquinic acid derivative 7	19.59	311	0.122 ± <0.001 ^a^	0.397 ± 0.014 ^b^	0.168 ± 0.003 ^c^	0.585 ± 0.006 ^d^	0.471 ± 0.030 ^e^
13	3-*O*-Feruloylquinic acid	22.61	255, 297 sh, 323	-	-	0.171 ± 0.005 ^a^	-	0.352 ± 0.046 ^b^
14	*p*-Coumaric acid	24.37	309	0.290 ± <0.001	-	-	-	-
15	Apigenin-6,8-di-*C*-glucoside	24.93	270, 334	-	0.335 ± 0.003 ^a^	1.87 ± 0.08 ^b^	2.63 ± 0.05 ^c^	2.83 ± <0.01 ^d^
16	*p*-Coumaric acid isomer	25.46	309	0.278 ± <0.001	-	-	-	-
17	*p*-Coumaroylquinic acid derivative 8	27.43	314	1.29 ± <0.01	-	-	-	-
18	Ferulic acid	28.94	256, 290 sh, 323	0.291 ± 0.002	-	-	-	-
19	Hydroxycinnamic acid 1	31.30	255, 299 sh, 327	1.70 ± 0.01	-	-	-	-
20	Apigenin-8-*C*-glucoside	33.69	267, 335	-	0.470 ± <0.001 ^a,b^	0.438 ± 0.012 ^a^	0.539 ± 0.01 ^b^	1.10 ± 0.08 ^c^
21	Kaempferol derivative 1	34.08	266, 347	20.1 ± 0.4	-	-	-	-
22	Kaempferol derivative 2	39.31	265, 346	6.26 ± 0.24	-	-	-	-
23	Apigenin-6-*C*-glucoside	41.30	269, 335	-	0.456 ± 0.024 ^a^	0.408 ± <0.001 ^b^	0.478 ± <0.001 ^a^	0.831 ± 0.027 ^c^
24	Quercetin-3-*O*-glucoside	43.34	257, 265 sh, 355	-	1.12 ± <0.01 ^a^	2.15 ± 0.04 ^b^	5.80 ± <0.01 ^c^	3.75 ± <0.01 ^d^
25	Quercetin-3-*O*-rutinoside	44.00	257, 265 sh, 256	21.0 ± 0.1 ^a^	3.61 ± <0.01 ^b^	0.539 ± 0.021 ^c^	1.24 ± 0.02 ^d^	2.54 ± 0.01 ^e^
26	Quercetin derivative 1	44.94	257, 265 sh, 355	-	1.08 ± 0.02 ^a^	8.23 ± 0.03 ^b^	7.33 ± 0.04 ^c^	6.98 ± 0.02 ^d^
27	Kaempferol-3-*O*-glucoside	47.41	265, 347	-	0.572 ± 0.002 ^a^	1.58 ± 0.10 ^b^	1.86 ± 0.01 ^c^	2.05 ± 0.01 ^d^
28	Kaempferol-3-*O*-rutinoside	48.17	265, 345	3.48 ± 0.09 ^a^	0.591 ± 0.002 ^b^	0.201 ± 0.049 ^c^	0.220 ± 0.005 ^c^	0.870 ± 0.002 ^d^
29	Kaempferol derivative 3	49.49	265, 347	-	-	4.74 ± 0.09 ^a^	2.48 ± 0.01 ^b^	3.55 ± 0.03 ^c^
30	Acylated kaempferol 1	51.28	266, 317, 355 sh	1.13 ± 0.02	-	-	-	-
31	Acylated kaempferol 2	54.70	267, 320, 357 sh	1.30 ± 0.01	-	-	-	-
	Total			59.6	14.2	30.5	37.9	35.8

* Results are expressed as mean ± standard deviation (SD) of three determinations. Different lowercase letters indicate the results are expressed as mean ± standard deviation (SD) of three determinations. Within lines, homogeneous groups are shown by the same letter (ANOVA followed by Tukey’s test) for *p* < 0.05.

**Table 5:** Quantification of Pb and Cd in aqueous moringa extracts (*n* = 6).

Sample	Pb (mg/kg Dry Extract)	Cd (mg/kg Dry Extract)
1	0.108 ± 0.011 ^a^	0.115 ± <0.001 ^a^
2	1.46 ± 0.12 ^b^	0.125 ± 0.002 ^a^
3	0.586 ± 0.043 ^c^	0.702 ± 0.031 ^b^
4	0.497 ± 0.032 ^c^	0.275 ± 0.035 ^c^
5	1.35 ± 0.09 ^d^	0.123 ± 0.005 ^a^

Within columns, homogeneous groups are shown by the same letter (ANOVA followed by Tukey’s test) for *p* < 0.05.

**Table 6:** Terms associated with tea consumption, reading labels during tea purchase, and number of citations from each gender.

Terms Associated with Tea Consumption		
	Citations by Women	Citations by Men
Leisure	24 (+)	3 (−)
Health	45	28
Diet	20	6
Wellness	44	31
Relaxation	43	23
Family Moments	21	5
Sophistication	3	0
Reading Labels		
	Citations by Women	Citations by Men
Title/Name	9	3
Type/Flavor	15	8
Brand	6	2
Production Site	4	5
Validity	6	3
Ingredients	25	11
Health Benefits	30	16
Price	9	8
I Do Not Notice	3 (−)	17 (+)

(+) or (−): the observed value is higher or lower than the expected theoretical value (*p* < 0.05) according to the Chi-square test.

## Data Availability

Data is contained within the article.
